# A Design of Adaptive Control and Communication Protocol for SWIPT System in 180 nm CMOS Process for Sensor Applications

**DOI:** 10.3390/s21030848

**Published:** 2021-01-27

**Authors:** Muhammad Riaz Ur Rehman, Imran Ali, Danial Khan, Muhammad Asif, Pervesh Kumar, Seong Jin Oh, Young Gun Pu, Sang-Sun Yoo, Keum Cheol Hwang, Youngoo Yang, Dong In Kim, Kang-Yoon Lee

**Affiliations:** 1 Department of Electrical and Computer Engineering, Sungkyunkwan University, Suwon 16419, Korea; riaz@skku.edu (M.R.U.R.); imran.ali@skku.edu (I.A.); danialkhan@skku.edu (D.K.); m.asif@skku.edu (M.A.); itspervesh@skku.edu (P.K.); geniejazz@skku.edu (S.J.O.); hara1015@skku.edu (Y.G.P.); khwang@skku.edu (K.C.H.); yang09@skku.edu (Y.Y.); dikim@skku.ac.kr (D.I.K.); 2 Department of Smart Automobile, Pyeongtaek University, Pyeongtaek 17869, Korea; rapter@kaist.ac.kr

**Keywords:** ASK, digital controller, energy harvesting, protocol, simultaneous wireless information and power transfer (SWIPT)

## Abstract

This paper presents an adaptive control and communication protocol (ACCP) for the ultra-low power simultaneous wireless information and power transfer (SWIPT) system for sensor applications. The SWIPT system-related operations depend on harvested radio frequency (RF) energy from the ambient environment. The necessary power for SWIPT system operation is not always available and it depends on the available RF energy in the ambient environment, transmitted RF power from the SWIPT transmitter, and the distance from the transmitter and channel conditions. Thus, an efficient control and communication protocol is required which can control the SWIPT system for sensor applications which mainly consists of a transmitter and a receiver. Multiple data frame structures are used to optimize the exchange of bits for the communication and control of the SWIPT system. Both SWIPT transmitter and receiver are capable of using multiple modulation schemes which can be switched depending on the channel condition and the available RF energy in the ambient environment. This provides support for automatic switching between the time switching scheme and power splitting scheme for the distribution of received RF power in the SWIPT receiver. It also adjusts the digital clock frequency at the SWIPT receiver according to the harvested power level to optimize the power consumption. The SWIPT receiver controller with ACCP is implemented in 180 nm CMOS technology. The RF frequency of the SWIPT operation is 5.8 GHz. Digital clock frequency at the SWIPT receiver can be adjusted between 32 kHz and 2 MHz which provides data rates from 8 to 500 kbps, respectively. The power consumption and area utilization are 12.3 µW and 0.81 mm².

## 1. Introduction

With the development of 5G, the use of wireless communication devices is enormously increased to make lives more comfortable and improve the quality of service [1,2,3,4]. This results in the development of internet of things (IoT) devices in which a massive number of wireless devices including sensors communicate with each other at much higher data rates and require an ultra-low power design for long-lasting operation [5,6,7]. In most cases, the sensor nodes operate on batteries and depend on the application such as a toxic environment, or inside the human body or the wall, where it is difficult to replace the batteries [8,9,10]. In addition, it requires significant resources for maintaining billions of IoT devices, providing batteries for the operation and the proper battery disposal described in [11,12,13]. Thus, an efficient energy harvesting and saving mechanism is required to reduce the overall power consumption of the IoT devices and intelligently harvest energy to minimize the dependency on batteries for the operation [14,15,16,17,18,19]. In energy harvesting, multiple energy sources which are wasted in the ambient environment are captured and converted into electrical energy. The sources for the energy harvesting for an IoT device may be solar, vibration, heat, wind or radio frequency [20]. Wireless power transfer (WPT) is mainly focused on short distance energy transmission than long distance. WPT has multiple limitations like high initial cost, distance constraints, maintaining field strength within safety limits and the use of a high frequency for energy transmission [21]. Since wireless nodes are usually at great distances from the base station, long-distance power and data communication techniques must be improved. The harvesting radio frequency (RF) energy from the received signal is an attractive alternative to solving the battery problems of IoT devices. This shows the advantage of continuous energy harvesting and stable performance in different kinds of RF environments [22,23]. The simultaneous wireless information and power transfer (SWIPT) allows to transfer power and information through same RF signal [24,25,26]. This technique can increase the life span of the battery or eliminate the need for a battery in wireless sensors. By transmitting both power and information in the SWIPT, this provides significant gains in terms of spectral efficiency, transmission delay, interference management and power consumption [27]. Several wireless power transfer protocols have been proposed for wireless sensor networks. A protocol based on an energy adaptive medium access control (EA-MAC) was proposed in [28]. This controls the duty cycle of the sensor nodes depending on the contention time and energy harvesting (EH) rate of each node. This requires a centralized control for the coordination to control the duty cycle of the sensor nodes. Another protocol, an on-demand MAC (ODMAC), was proposed in [29]. In this protocol, sensor nodes adjust their duty cycle to obtain an energy neutral (ENO) state where the harvested energy is greater than the consumed energy. In [30], an energy harvested receiver-initiated MAC (ERI-MAC) protocol was presented. A receiver node senses the channel to prevent collisions and a sender uses the channel when it receives a beacon from the receiver. In [31], a p-persistent MAC protocol was proposed for EH in machine-to-machine (M2M) networks. A frame is divided into four periods: a notification period (NP), a harvesting period (HP), a contention period (CP) and a transmission period (TP). The base station broadcasts a notification message during the NP and active devices prepare to harvest energy during the HP. Active devices harvest energy during the HP and participate in the contention during the CP. Finally, the devices which succeed during CP can transmit data during the TP. The protocols [29,30,31] consider external energy sources which do not affect the data transmission performance. In [32], an RF EH wireless sensor networks (WSNs) (RF-MAC) system was proposed where the same radio band was used for the transfer of energy and data. When a node needs energy, it broadcasts a request for energy (RFE) packet after which the transmitter starts providing energy pulses. However, in [32], the energy and data transmissions are independently performed which affect each other’s respective performance. To cope with this problem, a harvest-then-transmit scheme was introduced, in which a sensor node harvests energy from the hybrid access point (HAP) before the data transmission [33]. In [34], a modified EDCF MAC (HE-MAC) which is based on harvest then transmission. It increases the efficiency of wireless powered sensor nodes (WPSN) by managing the RF energy transmission and data transmission. In HE MAC, the energy and data transmission is not concurrently happening at the HAP or node. An energy-neutral control algorithm was proposed in [35]. When a sensor node has less stored energy, the protocol provides more power to the sensor nodes through the power beacon and the sensor nodes reduce their duty cycle to minimize the power consumption. As it updates the duty ratio in every frame, it then makes it less efficient. In [36], a design of duty cycle-based dual mode SWIPT receiver is presented. It is the PCB-based implementation of SWIPT with commercial devices which is not optimized for SWIPT operation. It uses a protocol to switch between single tone and multiple data demodulation based on the received RF energy. Mostly protocols consider the time switching (TS) scheme for the SWIPT. This scheme is only beneficial when the harvested energy is not available for the simultaneous operation of energy harvesting and information decoding. When harvested energy in the ambient environment is sufficient for the simultaneous operation of energy harvesting and information decoding then the power splitting (PS) scheme is used to enhance the performance and efficiency of the SWIPT system. Due to the limited energy available for the SWIPT system, an efficient energy utilization scheme is necessary considering the challenging scenarios of the SWIPT system [37]. RFID and SWIPT share the structural similarities such as RF energy harvesting and digital communication [38,39,40]. The source of energy harvesting for RFID is based on a dedicated RF transmitter/source, unlike the SWIPT, where the source of energy harvesting is mainly based on RF energy in the ambient environment. However, in some cases, where a transmitter in SWIPT is transmitting data and power to the receiver, this scenario is similar to RFID operation. Various operational challenges and improved energy harvesting techniques related to SWIPT-enabled wireless sensor network are discussed in [41,42,43]. The bit error rate (BER) performance of binary differential phase-shift keying (BDPSK) modulated the signal with a nonlinear EH model for a sensor node was investigated in [41]. This shows that the linear EH model is not suitable for practical SWIPT application in a sensor network. An energy-aware SWIPT routing algorithm is proposed in [42] for a multi-hop energy constrained wireless network. This allocates the information and energy in relation to the allocation algorithm during the path finding process. A comprehensive overview of the integration of SWIPT and cooperative relay (CoR) techniques for next generation wireless communication system is provided in [43]. This provides a detailed discussion about various architectures, resource allocation schemes and relay selection algorithms for SWIPT–CoR in a sensor network. This paper presents an adaptive control and communication protocol which allows for adaptive switching between the time-switching scheme and the power-splitting scheme based on the RF energy available in the ambient environment. Moreover, it also adaptively adjusts the digital clock frequency at SWIPT receiver to further enhance the SWIPT operation in case sufficient ambient energy is available. Additionally, it provides support for automatically controlling SWIPT communication by switching between low power frames and high power frames according to the harvested energy level at the SWIPT receiver. The major contributions of this paper are as follows:

- A control and communication protocol for an ultra-low power SWIPT system for sensor application.
- Adaptive switching between the time-switching scheme and the power-splitting scheme based on the RF energy available in the ambient environment.
- Adaptive control of digital frequency at the SWIPT receiver based on the available harvesting energy.
- Centralized control of the SWIPT system which is controlled by the SWIPT transmitter.
- Adaptive data frame selection to improve the communication in a low ambient energy scenario.

The rest of the paper is structured as follows: in Section 2, the proposed SWIPT system architecture is discussed. Section 3 presents the proposed adaptive control and communication protocol. The experimental results are discussed in Section 4. Lastly, the paper is concluded in Section 5.

## 2. Proposed SWIPT System Architecture

### 2.1. SWIPT Transmitter

**Figure 1:**
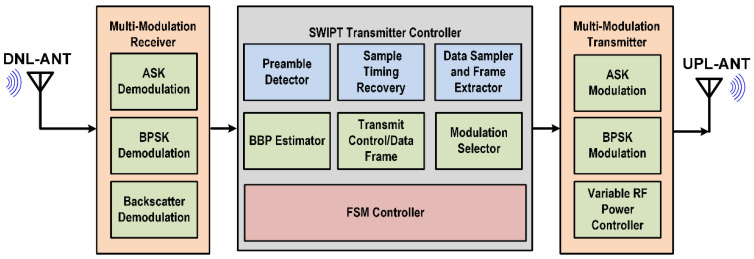
Architecture of the proposed simultaneous wireless information and power transfer (SWIPT) transmitter with the adaptive control and communication protocol (ACCP).

The proposed architecture of the SWIPT transmitter is shown in Figure 1. It mainly consists of three main blocks which are multi-modulation receiver (MMR), SWIPT transmitter controller (STC), and the multi-modulation transmitter (MMT). The MMR supports multiple modulations that can be used depending on the channel condition and harvesting power available at the receiver. In the proposed SWIPT system architecture, the STC acts as a SWIPT system controller which takes decisions according to the energy harvesting status at the SWIPT receiver. Its main goal is to maximize energy transfer while keeping high data communication throughput. Due to adequate power at the transmitter, STC is controlling the overall operation of SWIPT to increase reliability. The STC consists of multiple blocks to control the SWIPT operation and performs communication. The preamble detector detects the preamble in the received data. The sample timing recovery block recovers the sampling timing from the preamble in the received data. The data sampler and frame extractor extracts the bytes from the received data and classifies the received frame. The boost burst period (BBP) estimator block momentarily increases the RF power to allow for the fast charging of supercapacitor at the SWIPT receiver. The finite state machine (FSM) controller controls the operations in the STC according to the adaptive control and communication protocol (ACCP). The modulation selector block selects the modulation depending on the channel condition and energy harvesting at the SWIPT receiver. The SWIPT transmitter has a variable RF power controller for controlling transmitted RF power. The uplink antenna (UPL-ANT) is used to transmit the RF power and data to the SWIPT receiver. A downlink antenna (DNL-ANT) is used to receive the data signals from the SWIPT receiver.

### 2.2. SWIPT Receiver

**Figure 2:**
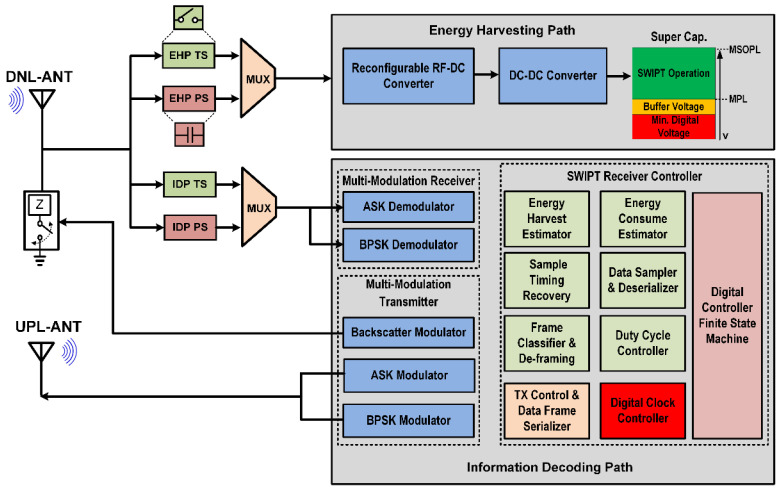
Architecture of the proposed SWIPT receiver with the ACCP.

**Figure 3:**
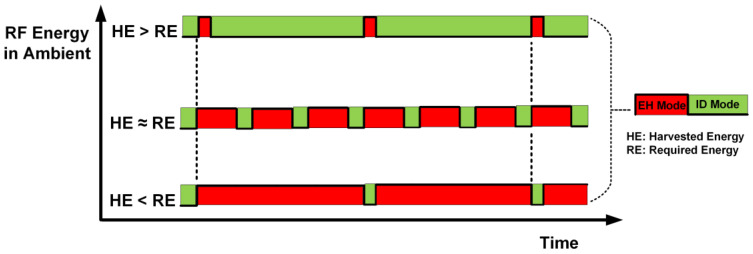
SWIPT receiver duty ratio depending on the RF energy in the ambient environment.

The proposed architecture for the SWIPT receiver is shown in Figure 2. It mainly consists of three main sections, which are the energy harvesting path (EHP), the information decoding path (IDP), and the SWIPT receiver controller (SRC). This architecture supports both time-switching and power-splitting approaches to distribute the received RF signal among the EHP and IDP. The EHP consists of a reconfigurable RF-DC converter, DC-DC converter and a supercapacitor. The RF-DC converter performs the conversion of received RF power into DC voltage. The output of the RF-DC is not regulated and varies with the received amount of RF energy. To stabilize the DC output and provide sufficient voltage for the operation of other blocks at the SWIPT receiver, a DC-DC converter was used. The harvested energy was stored in supercapacitor. This provides energy for the IDP and SRC. The received data and control instructions are decoded in the IDP block. This performs the task of data communication including reception and transmission. The received data or instruction is processed by the SRC. The IDP supports multiple modulation schemes for the reception and transmission of the data. The SRC is the main processing and controlling block in the SWIPT receiver. It consists of multiple blocks to perform SWIPT operations at the SWIPT receiver. The energy harvesting estimator monitors the amount of available energy in the supercapacitor. The energy consumption estimator (ECM) calculates the power consumption for receiving, processing and transmitting date. It helps the SWIPT receiver perform operations only when sufficient energy is available for the task. The sample timing recovery block recovers the sampling frequency from the preamble to correctly sample the received data. The data sampler and deserializer block extracts the data bytes and these data bytes are further classified by the frame classifier and de-framing block. The duty cycle controller controls the switching between the EHP and IDP during the time-switching scheme. The SWIPT receiver can switch between the time-switching and power-splitting schemes according to the harvested energy available at the SWIPT receiver. The digital controller finite state machine is responsible for controlling the overall operations at the SWIPT receiver according to the ACCP. Due to the limited available energy at the SWIPT receiver, the preferred way of data transmission from the SWIPT receiver is backscattering. However, if the available energy is sufficient, then the other modulation schemes can be used for data transmissions such as the ASK or BPSK modulation. The digital clock controller (DCC) adaptively adjusts the clock frequency for the SRC based on the harvested energy at the SWIPT receiver. Figure 3 shows the varying duty cycle selected by the SWIPT transmitter which depends on the available RF energy in the ambient environment for the SWIPT receiver.

## 3. Proposed Adaptive Control and Communication Protocol

**Figure 4:**
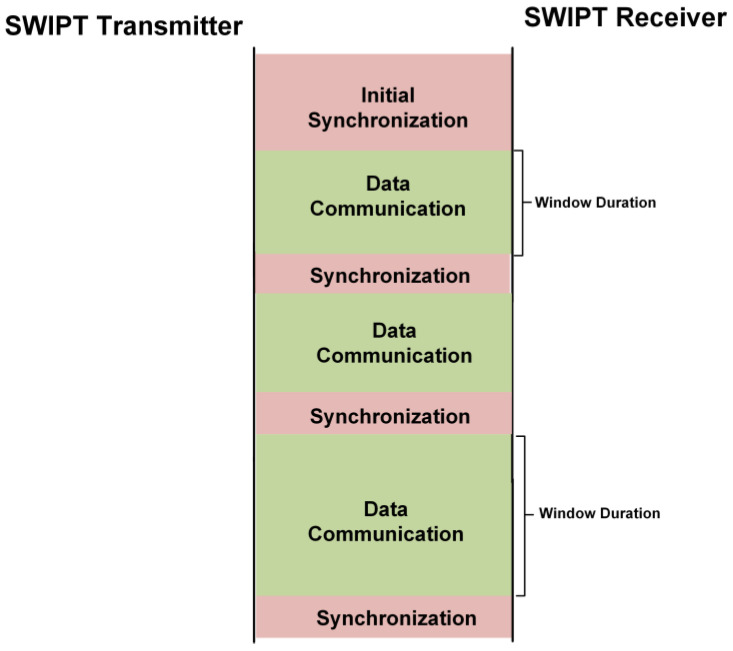
Top operation of the ACCP in SWIPT system.

**Figure 5:**
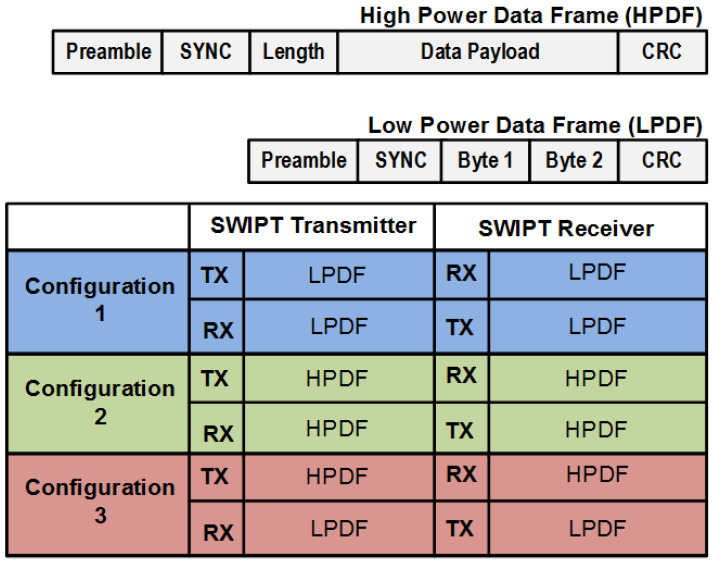
Frame structure for data packet communication (DPC) and its configuration in the SWIPT system.

The top operational flow diagram is shown in Figure 4. Two main tasks are performed between the SWIPT transmitter and the SWIPT receiver, which are synchronization and data communication. During synchronization, control frames are exchanged between the SWIPT transmitter and the SWIPT receiver for the adaptive control of the overall SWIPT operation based on the energy harvesting condition at the SWIPT receiver. During the data communication period, data frames are exchanged between the SWIPT transmitter and the SWIPT receiver. The data communication is performed for the period of window duration. The length of window duration is selected based on the energy harvesting situation at the SWIPT receiver. Initial synchronization is performed based on the default SWIPT parameters. The default SWIPT parameters are optimized for the maximum energy harvesting at SWIPT receiver. This ensures that sufficient energy is available to perform data processing and transmission operations. The SWIPT transmitter and the SWIPT receiver support low power and high-power frames for data packet communication (DPC). The main purpose is to minimize the number of bits for transmitting, processing and receiving in the case of low harvesting power available at the SWIPT receiver. The frame structure is shown in Figure 5.

The low power data frame (LPDF) and high power data frame (HPDF) consist of a preamble, sync, type of frame, number of bytes, data bytes and cyclical redundancy check (CRC). The difference between the high and low power frames is a greater number of data bytes in the high power frame. In the case of the LPDF, the bytes field number is skipped and only two data bytes are transmitted. In the case of HPDF, the bytes field number indicates the number of bytes available in the data byte field. Three configurations can be used in the SWIPT system:

- SWIPT transmitter sends and receives a low power frame.
- SWIPT transmitter sends a high power frame and receives a high power frame.
- SWIPT transmitter sends a high power frame and receives a low power frame.

These configurations reduce the power consumption at the SWIPT receiver. In the first configuration, the receiver is harvesting less energy, which is not sufficient for the receiving and transmitting HPDF. In the second configuration, when the harvesting energy is sufficient, then both the SWIPT transmitter and receiver can communicate through the HPDF. In the third configuration, the harvesting energy at the SWIPT receiver is sufficient for receiving high data frames but not for the transmission of HPDF. This configuration is trying to optimize the data transmission depending on the energy available at the SWIPT receiver.

### 3.1. Role of SWIPT Transmitter

**Figure 6:**
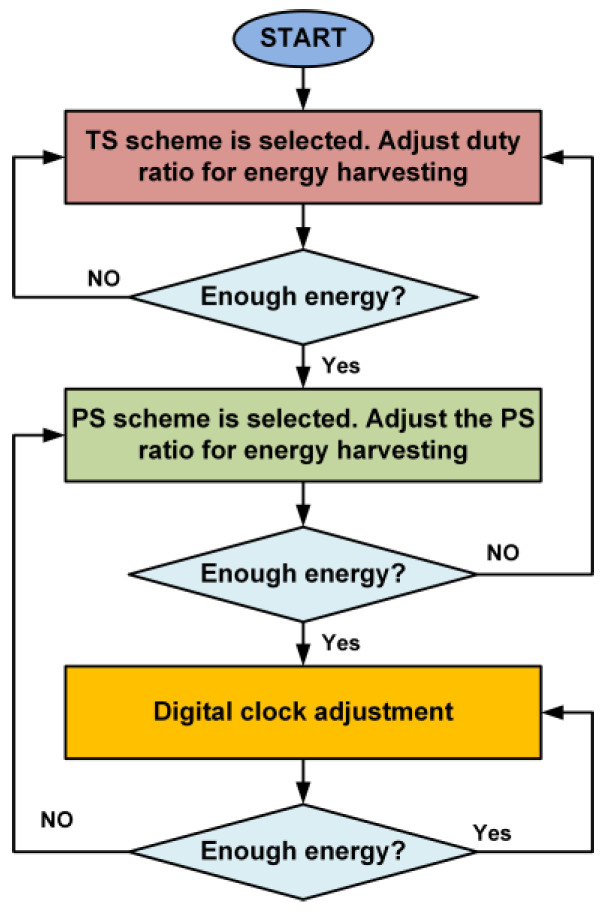
Flow diagram for the selection of the TS scheme, PS scheme and digital clock adjustment at the SWIPT receiver.

In the proposed design of the SWIPT system, the SWIPT transmitter acts as a master and the SWIPT receiver’s role is that of a slave. For the SWIPT system optimization, the STC controls the communication, RF power splitting scheme, energy harvesting duty cycle and digital clock frequency based on the harvested energy at the SWIPT receiver. Similarly, depending on the harvesting energy level at SWIPT receiver, the SWIPT transmitter can adjust the RF transmitted power, the selection between the LPDF/HPDF along with the appropriate modulation schemes to optimize the performance of the energy harvesting and data communication at the SWIPT receiver. Figure 6 shows the flow diagram for the selection of the TS scheme, PS scheme, and digital clock adjustment at the SWIPT receiver.

Initially, the TS scheme is selected at the SWIPT receiver with the default setting for maximum energy harvesting. When the energy harvesting is sufficient, then the SWIPT transmitter adjusts the duty ratio accordingly. If the available energy is sufficient, then the SWIPT transmitter switches the RF power distribution from the TS to PS scheme. This takes advantage of the sufficient RF energy available in the ambient environment and this improves the SWIPT operation performance. The PS ratio is also adjusted by the SWIPT transmitter based on energy harvesting at the SWIPT receiver. If the available RF energy is still sufficient, then the SWIPT transmitter performs the digital clock adjustment to increase the performance of the data communication. This increases or decreases the digital clock frequency based on the available RF energy at the SWIPT receiver.

**Figure 7:**
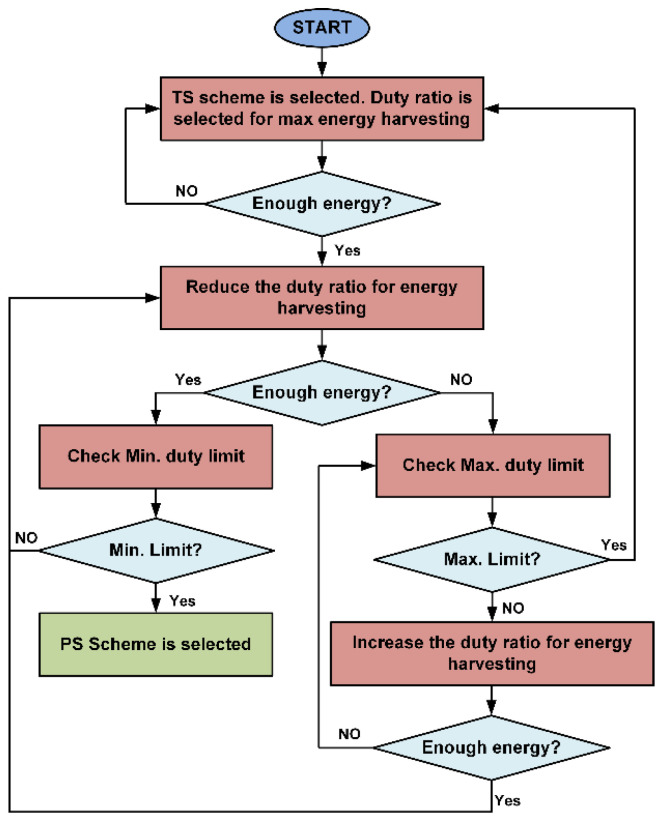
Flow diagram for the SWIPT transmitter during the TS scheme.

**Figure 8:**
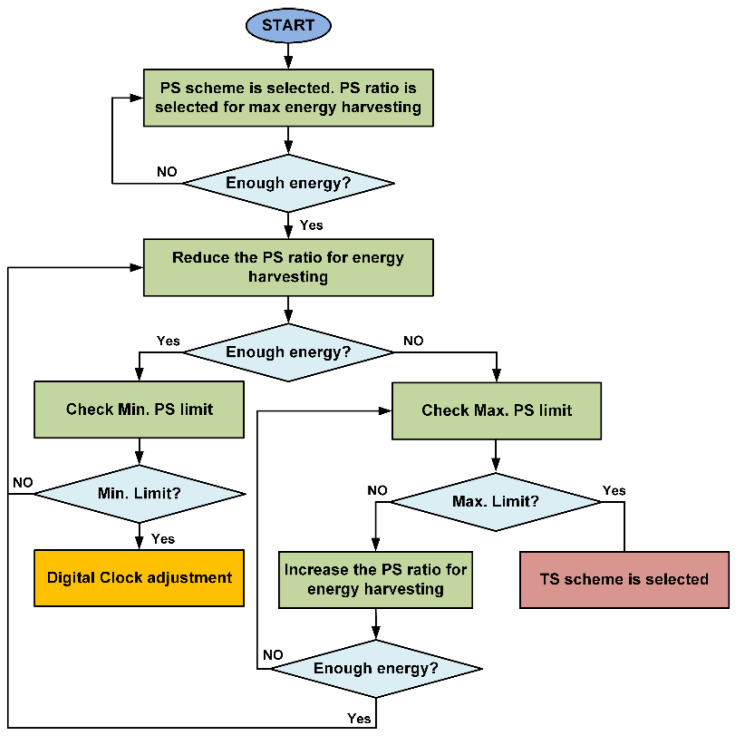
Flow diagram for the SWIPT transmitter during the PS scheme.

Figure 7 shows the operations performed by the SWIPT transmitter during the TS scheme. At the start, the TS scheme is selected with a duty ratio for maximum energy harvesting. When the SWIPT receiver is powered up, then based on energy-harvesting information provided by the SWIPT receiver, the SWIPT transmitter increases or decreases the duty ratio. If due to better energy harvesting conditions, the SWIPT transmitter switches to the PS scheme when it reaches the minimum duty ratio for energy harvesting. Similarly, if the energy harvesting condition becomes worse, then the SWIPT transmitter increases the duty ratio up to a maximum limit for energy harvesting. Figure 8 shows the tasks performed by the SWIPT transmitter during the PS scheme.

**Figure 9:**
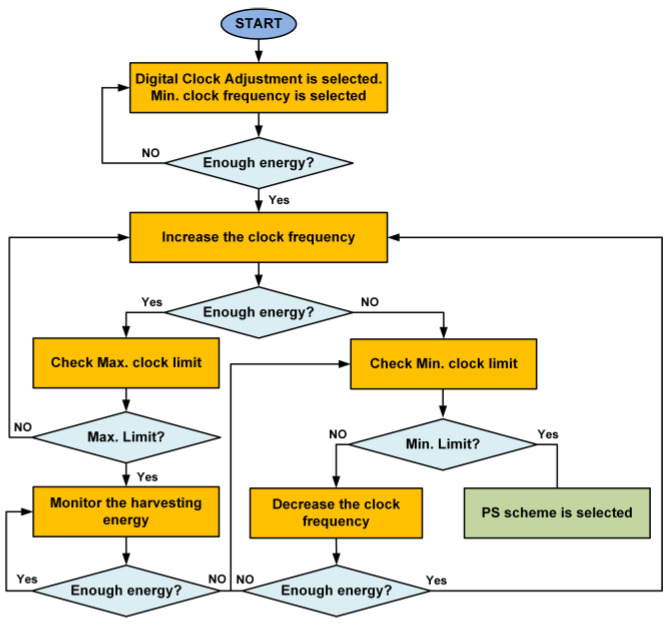
Flow diagram for the SWIPT transmitter during the digital clock adjustment.

Initially, the PS ratio is selected for maximum energy harvesting for the SWIPT receiver. If data communication performs adequately and the available energy is sufficient then the PS ratio for energy harvesting is decreased to increase the data communication performance. The SWIPT transmitter selects the digital clock adjustment mode if it reaches the minimum PS ratio for energy harvesting. However, if the energy harvesting condition is not sufficient, then the SWIPT transmitter switches back to the TS scheme after reaching the maximum PS ratio for energy harvesting. Figure 9 shows the operations of SWIPT transmitter during the digital clock adjustment at the SWIPT receiver. If the ambient energy is sufficient, then the SWIPT transmitter will increase the digital clock of the SRC. This will increase data communication performance and higher data rates are possible. If the ambient RF energy is not sufficient, then the SWIPT transmitter switches back to the PS scheme to maintain the operation of the adaptive and optimized SWIPT.

### 3.2. Synchronization between SWIPT Transmitter and Receiver

**Figure 10:**
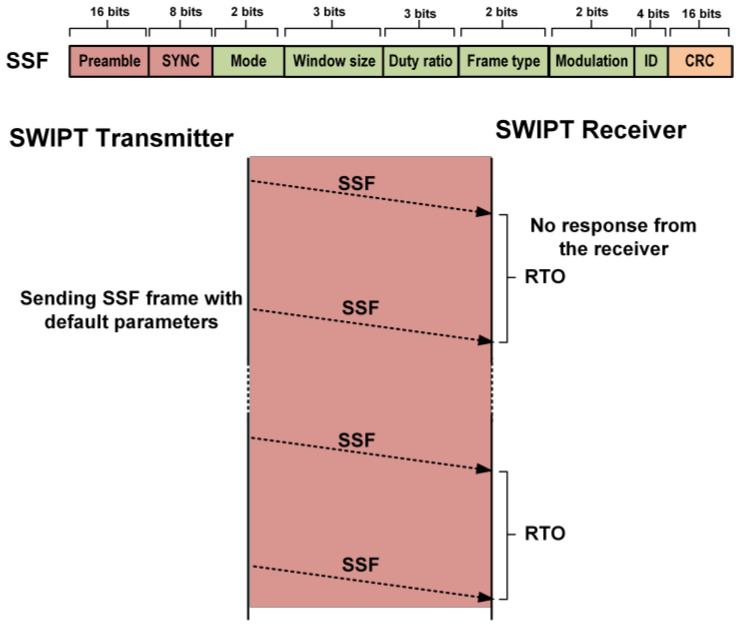
Transmission of the synchronization sequence frame (SSF) frame from the SWIPT transmitter.

At the start of the communication, the SWIPT transmitter needs to synchronize with the SWIPT receiver. The SWIPT transmitter sends the synchronization sequence frame (SSF) frame to the SWIPT receiver. The structure of the SSF frame is shown in Figure 10. It carries the information of the frame type, window duration, duty ratio, modulation and transmitter ID. The SWIPT transmitter keeps sending the SSF until the response from the SWIPT receiver is received. After sending the SSF frame, the SWIPT transmitter waits for a duration of a receive timeout period (RTO). In RTO, the SWIPT transmitter waits to receive a reply from the SWIPT receiver as an acknowledgment (ACK) frame. After RTO, the SWIPT transmitter sends the SSF frame again, which is shown in Figure 10. The SWIPT receiver initially has no power for the digital controller to control the SWIPT operation. Thus, the SWIPT receiver is designed to harvest RF energy to store energy in the supercapacitor. Initially, the only energy harvesting path is active to store sufficient energy to power up the SRC. When sufficient energy is available in the supercapacitor, which is above the minimum power limit (MPL) for the operation of the SRC, then the SRC is powered up and initializes the SWIPT receiver with default SWIPT parameters. The default control values are optimized for the maximum energy harvesting and minimum power consumption at the SWIPT receiver. The SWIPT receiver waits until the harvested energy is sufficient for receiving the data from the SWIPT transmitter. The SRC continuously monitors the supercapacitor’s energy level and when it reaches above the minimum SWIPT operation power limit (MSOPL), then it turns on the SWIPT receiver RX block to receive the data from the SWIPT transmitter. The SWIPT receiver is capable of automatically recovering a bit of the sampling clock and extract the transmitted information from the SWIPT transmitter.

**Figure 11:**
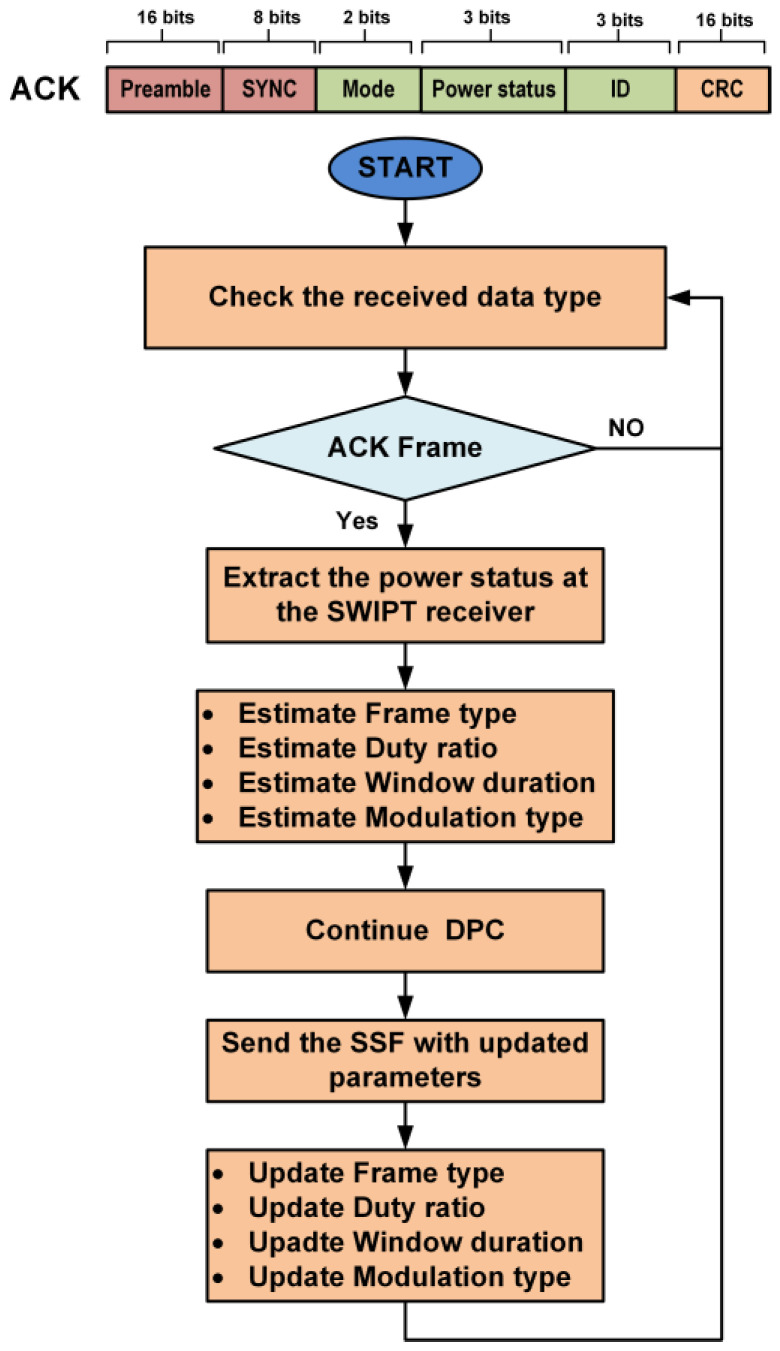
Structure of the acknowledgment (ACK) frame and the SWIPT control parameters updated at SWIPT transmitter.

The bit sampling clock is recovered by the preamble in the transmitted data. The SYNC is used to align the frame structure and extract the data byte information. After decoding the received data, the SWIPT receiver extracts the control and communication parameters. The selection of the LPDF/HPDF in the DPC is selected as per frame type field in SSF. The duty ratio for energy harvesting is adjusted according to the SSF. The window duration (WD) for the DPC is also updated by the SSF frame. The modulation scheme is also updated by the SWIPT receiver according to the SSF frame. The SWIPT receiver updates all internal control counters and signals so that it is synchronized with the SWIPT transmitter. When the required energy for transmission is available in the supercapacitor, then the SWIPT receiver transmits the ACK frame. The ACK frame contains the current selected mode, power status and SWIPT receiver ID, at the SWIPT receiver. The SWIPT transmitter receives the ACK frame from the SWIPT receiver. After decoding the ACK frame, it extracts the power status at the SWIPT receiver. The SWIPT transmitter estimates the new values of parameters like duty ratio, RF transmitted power level, window duration, frame type, and modulation type. The SWIPT transmitter applies these updated values after sending these values to the SWIPT receiver in the next SSF frame, as shown in Figure 11. When the SWIPT transmitter obtains the ACK signal from the SWIPT receiver, this selects the frame type and modulation schemes depending on the power status for energy harvesting.

### 3.3. Data Communication between the SWIPT Transmitter and SWIPT Receiver

**Figure 12:**
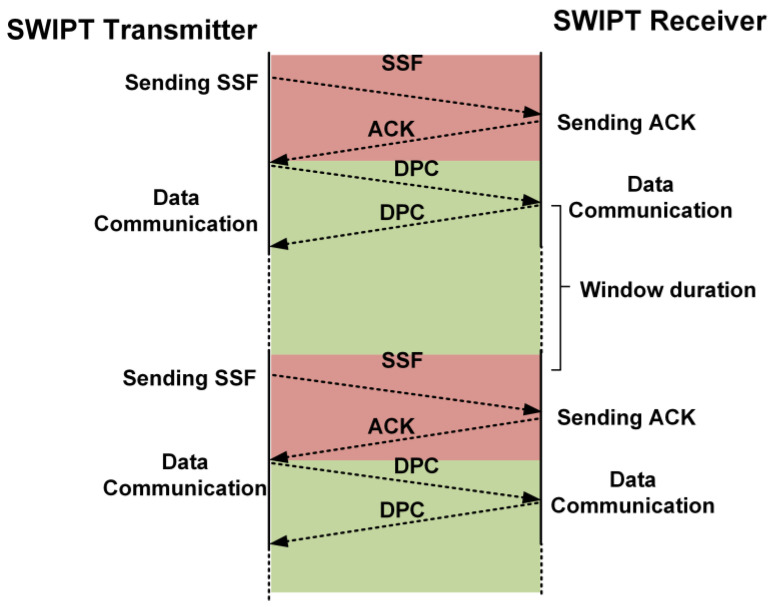
Exchange of the data frames for window duration after synchronization.

**Figure 13:**
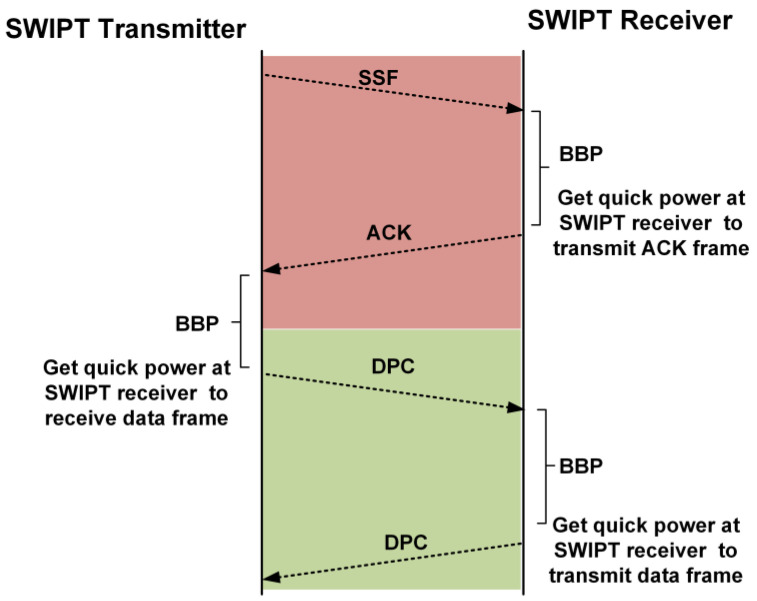
Transmission of the ACK frame after the BBP at the SWIPT receiver.

After the reception of the ACK frame from the SWIPT receiver, the SWIPT transmitter starts the exchange of data packets in the window duration. The number of data packet exchange is determined by the window duration. If the window duration value is five, then the number of data packet frames exchanged between the SWIPT transmitter and the receiver is five. Figure 12 shows the exchange of the data packet frame in the window duration. After the exchange of the required number of data frames in the DPC between the SWIPT transmitter and the SWIPT receiver, the SWIPT transmitter again sends the SSF for the updated communication and control parameters based on the last received ACK frame from the SWIPT receiver. After sending the SSF, the SWIPT transmitter configures itself based on the updated communication and control parameters. The SWIPT receiver decodes the SSF frame and applies the updated communication and control parameters. Then, it sends the ACK frame to the SWIPT transmitter. The transmitter receives the ACK frame based on updated communication and control parameters. Then, both the SWIPT transmitter and receiver start communicating with the updated communication and control parameters. The SWIPT transmitter can estimate the boost burst period (BBP) from the power status in the ACK frame to increase the performance of the SWIPT system. During BBP, it will temporarily switch to a maximum power transfer configuration. The BBP occurs especially just after when the SWIPT receiver completes the receive or transmit operation. Its main purpose is to quickly charge up the supercapacitor for the next operation. After this BBP, the SWIPT transmitter returns to its normal duty ratio and RF power which is decided based on the SWIPT receiver power status. The available power in the supercapacitor is utilized by the SWIPT receiver for receiving and processing the SSF frame. Due to limited harvesting power, the SWIPT receiver waits to acquire sufficient power to transmit the ACK frame to the SWIPT transmitter. The SWIPT transmitter’s BBP helps in the fast acquisition of power for the transmission of the ACK frame, as shown in Figure 13.

**Figure 14:**
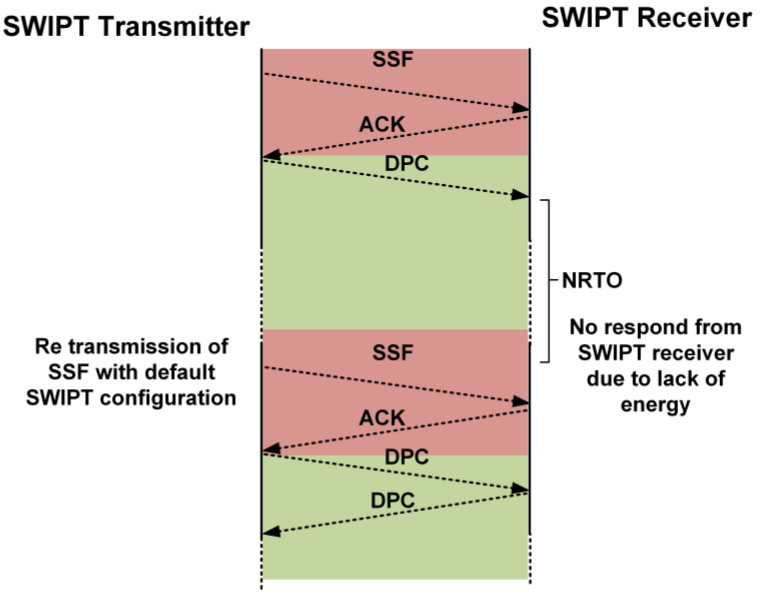
Re-synchronization by the SWIPT transmitter when there is no response from the SWIPT receiver.

If the SWIPT transmitter does not receive any response during synchronization or during DPC, then after the no response timeout period (NRTO), the SWIPT transmitter switches to default communication and control parameters and starts sending the SSF frame. The reason for the lack of response from the SWIPT receiver is the absence of a sufficient available energy level at the supercapacitor. Thus, the SWIPT transmitter needs to initialize the synchronization operation again between the SWIPT transmitter and the receiver, as shown in Figure 14.

### 3.4. Estimation of Received Power

The harvested power $P\left(E,P_{rx}\right)$ at the SWIPT receiver is given as

(1) $$P\left(E,P_{rx}\right)=\eta_{E}\left(E,P_{rx}\right).P_{rx}$$

where $P_{rx}$ is the received power and $\eta_{E}\left(E,P_{rx}\right)$ is the energy harvesting efficiency function with respect to the stored energy in the supercapacitor, which is given as

(2) ηE(E,Prx)=ηV(2EC,Prx)

where *E* is the energy stored in the supercapacitor having capacitance *C*. Energy stored in the supercapacitor is decreased due to the energy leakage *E_Leak_* which can be given as

(3) $$E_{Leak}=\frac{2}{C.R_{Leak}}.E$$

where *R_Leak_* is the leakage resistance of the supercapacitor. For the basic operation of the digital controller at the SWIPT receiver, a minimum energy *E_min_* is required which is given as

(4) $$E_{min}=\frac{C{\left(V_{min}\right)}^{2}}{2}$$

where *V_min_* is the minimum voltage required for the operation of a digital controller. The maximum energy *E_max_* that can be stored in a supercapacitor is given as

(5) $$E_{max}=\frac{C{\left(V_{max}\right)}^{2}}{2}$$

The SWIPT receiver continuously estimates the received harvesting power level and the amount of energy consumed for the data receiving, data processing, and data transmitting operations. The SWIPT receiver estimates the amount of harvested RF power by monitoring the supercapacitor’s charging rate. The faster it charges, the more the SWIPT receiver is harvesting RF energy.

**Figure 15:**
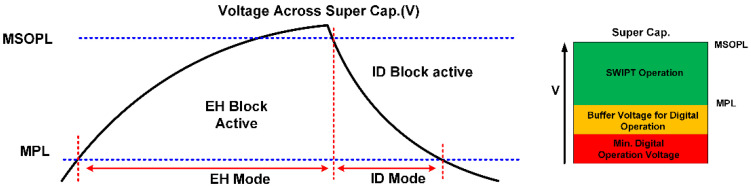
Estimation of the charging and discharging rate of the supercapacitor at the SWIPT transmitter and receiver.

**Figure 16:**
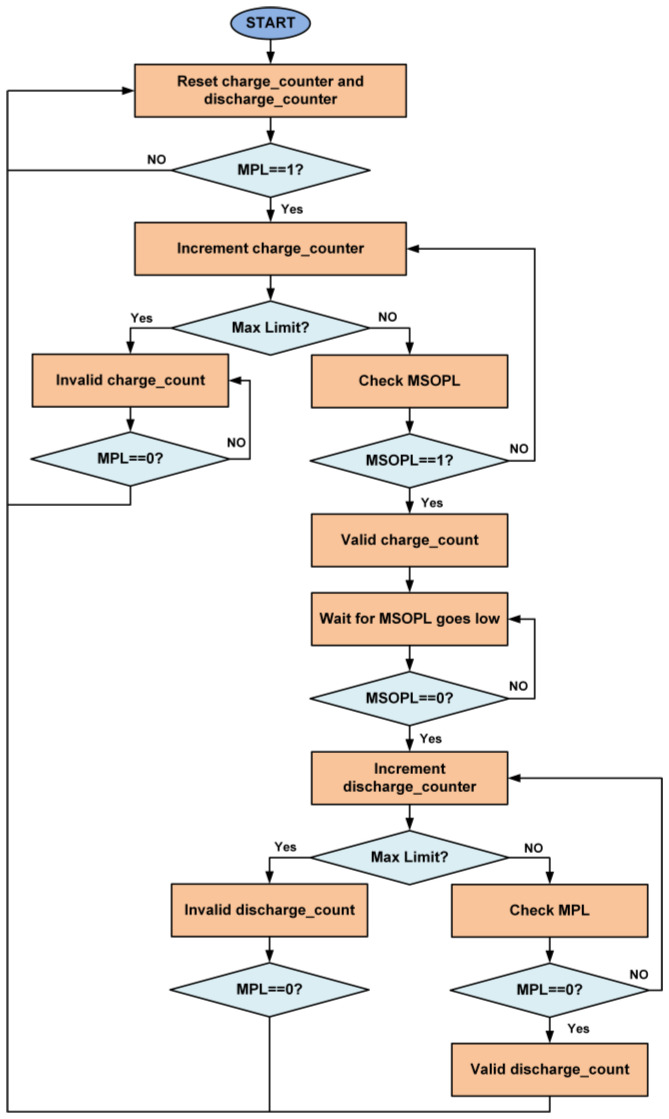
Flow diagram for the estimation of the charging and discharging rate of the supercapacitor at the SWIPT transmitter and receiver.

Similarly, it monitors the discharge rate of the supercapacitor. Figure 15 shows the limits of the voltage across the supercapacitor for the estimation of power status and power consumption. The flow diagram for the estimation of power status is shown in Figure 16. Two counters are used for the calculation of time duration, which are the charge counter and the discharge counter. When the energy level at the supercapacitor is above the MPL limit, then the charge counter will start incrementing. The controller waits until the energy level in the supercapacitor exceeds the MSOPL limit. When the energy in the supercapacitor reaches the MSOPL limit then the charge counter value is considered as valid and it is used in the power status calculation. If the energy in the supercapacitor cannot reach the MSOPL limit, then the charge counter will reach its maximum counting limit. In this situation, the charge counter value is considered as invalid and cannot be used in power status calculation. For the estimation of the rate of power consumption at the SWIPT receiver, the discharge counter is used. When the energy level drops below the MSOPL limit, the discharge counter starts incrementing. It will keep incrementing until the energy level in the supercapacitor drops below the MPL limit. This provides the valid discharge counter value for the calculation of power consumption while performing the SWIPT operation. If the discharge counter reaches its maximum counting limit, then it will provide an invalid discharge counter value which cannot be used for power consumption calculation.

## 4. Experimental Results

**Figure 17:**
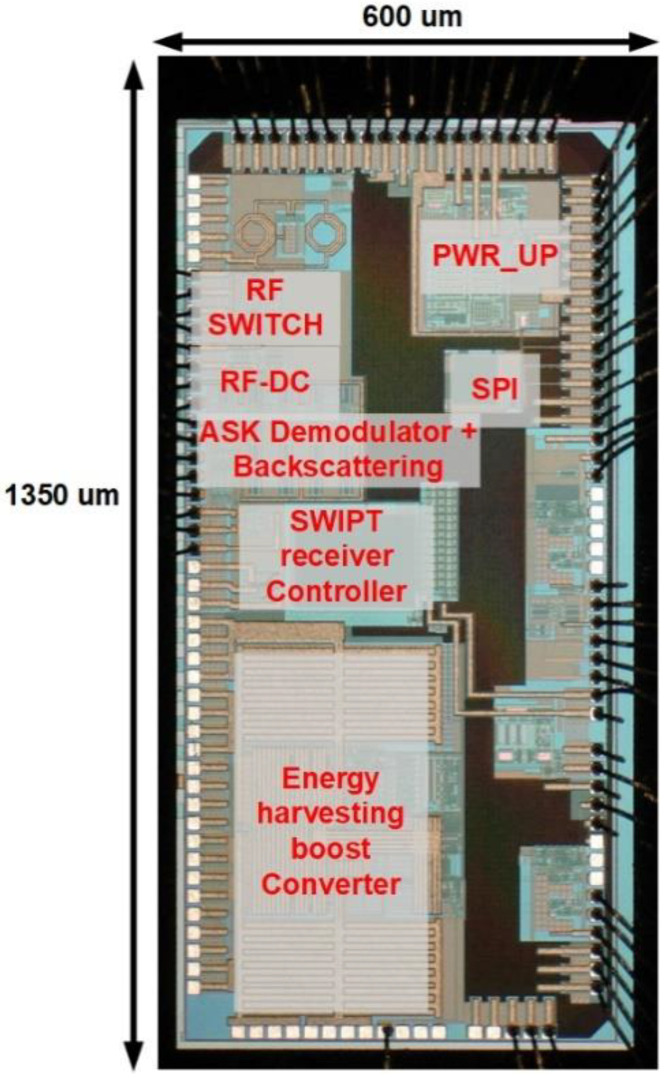
Chip photograph of the SWIPT IC.

**Figure 18:**
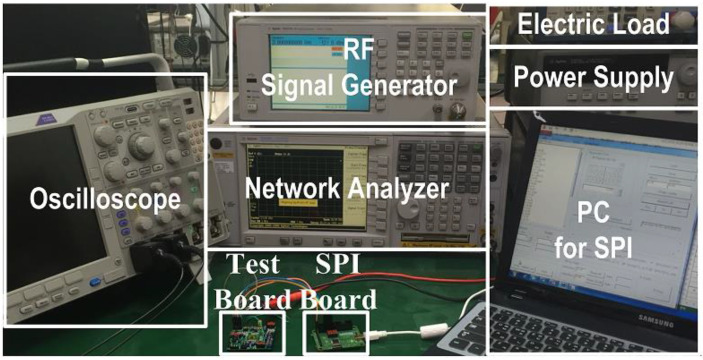
Measurement setup for the SWIPT IC.

The SWIPT receiver chip is designed and fabricated using a 180 nm CMOS process with an active area of 1350 µm × 600 µm. The chip includes an RF-DC converter, energy harvesting boost converter, RF switches, 2/4-ASK demodulator, backscattering circuit and a SWIPT receiver controller. The digital logic for the SWIPT receiver controller consumes 3.42 µW power and the area utilization is 0.0025 mm^2^. The chip photograph is shown in Figure 17. The measurement setup of the SWIPT is shown in Figure 18.

**Figure 19:**
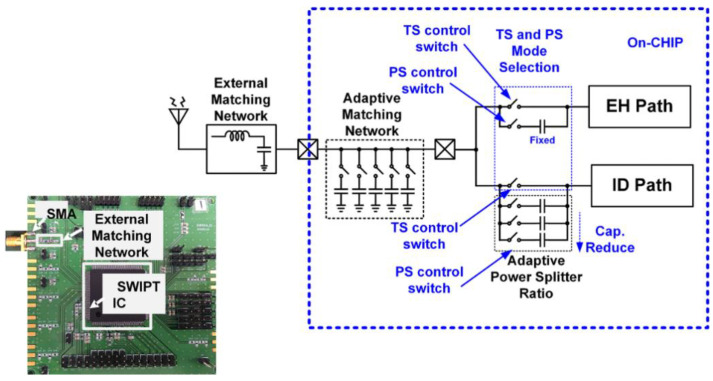
RF power transfer and matching network.

**Figure 20:**
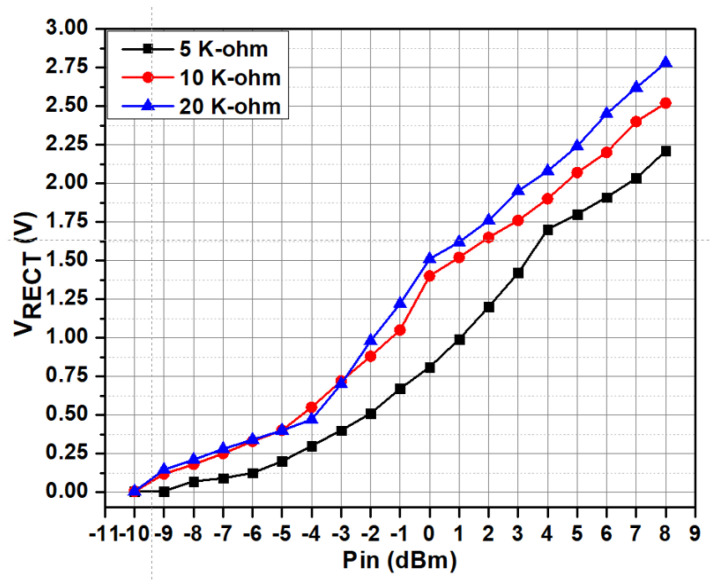
Output voltage of the RF-DC converter at multiple RF input power levels.

**Figure 21:**
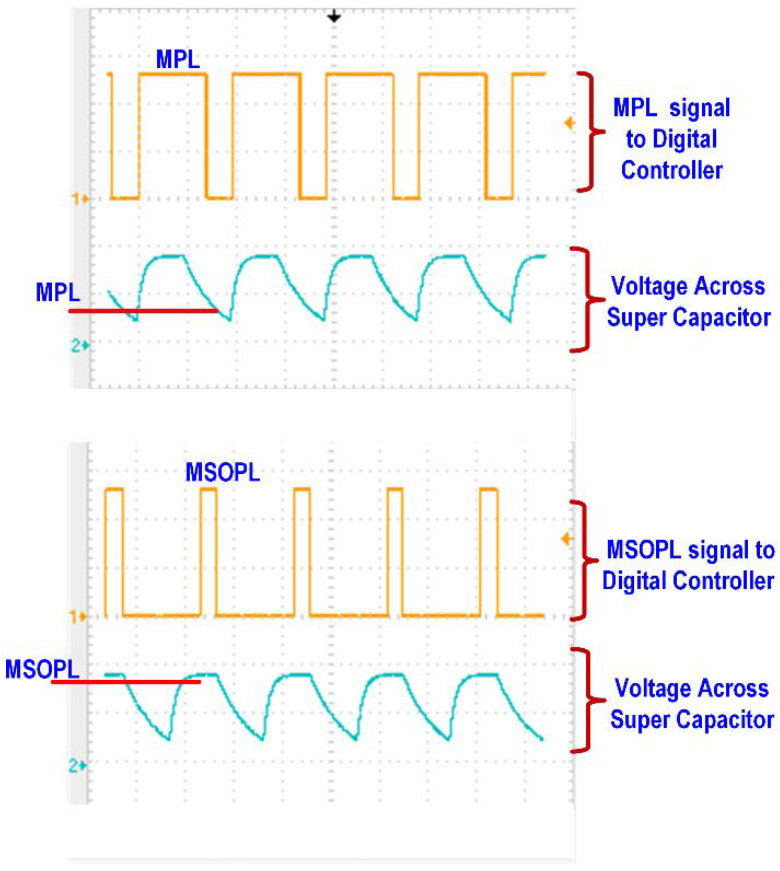
Charge and discharge voltage across the supercapacitor and MSOPL/MPL output signals.

**Figure 22:**
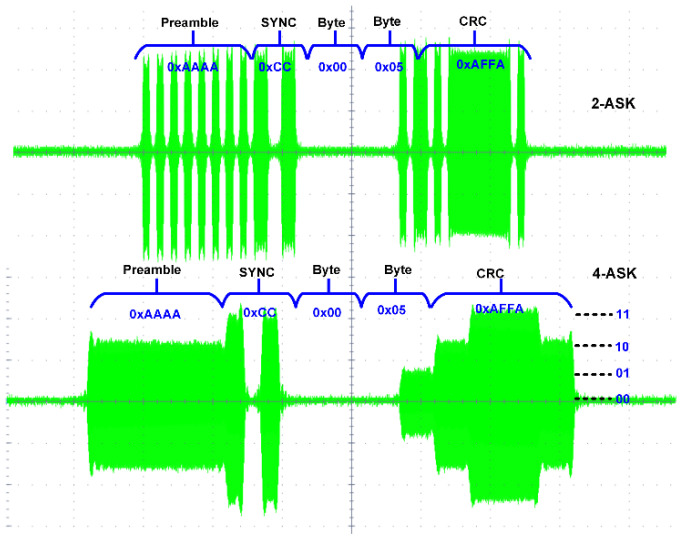
The RF 2-ASK and 4-ASK modulated SSF frame at 5.8 GHz.

**Figure 23:**
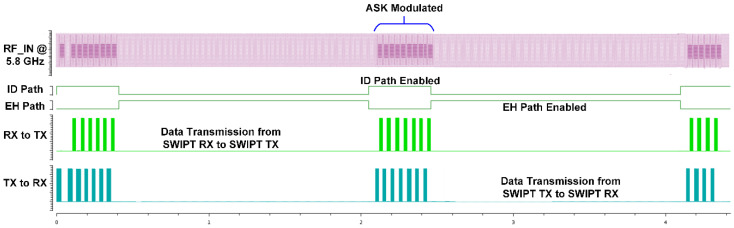
Data communication between the SWIPT transmitter and SWIPT receiver with a time switching scheme.

**Figure 24:**
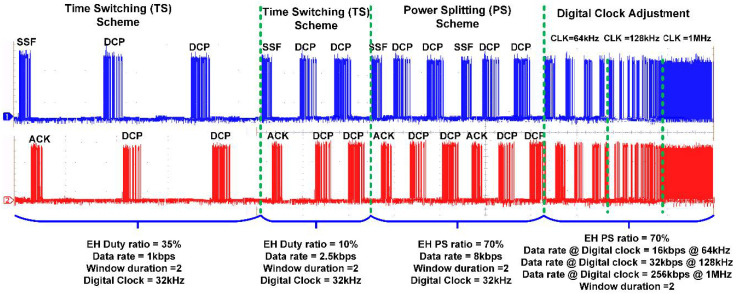
SWIPT system mode switching and effects on data rates.

The RF signal to be transmitted from the SWIPT transmitter is generated through the vector signal generator in which different modulation schemes can be selected. The RF signal is used by both the EH and ID path for energy harvesting and the information decoding, respectively. The TS and PS mode selection is performed by the RF switches which connects the received RF power to the EH path through the TS path or PS path. When the TS path is active, then the PS path stays open through the RF switch. The received RF power is distributed between the EH path and the ID path through duty cycle operation. When the PS path is active, then the TS path is disconnected by the RF switch. The received RF power is simultaneously transferred to the EH path and the ID path through the adaptive power splitter ratio, as shown in Figure 19. The output voltages of the RF-DC converter at different input RF power levels are shown in Figure 20. The output voltages are plotted with multiple load conditions. It can be shown that the output voltage of the RF-DC converter increases when the input RF power is increased. The harvested energy is stored in the supercapacitor which is used to provide power for information decoding and the digital controller. To measure the harvested power at the SWIPT receiver, the rate of charge and discharge of the supercapacitor is estimated. Two thresholds are used at the voltage across the supercapacitor, namely MSOPL and MPL. Figure 21 shows the voltage across the supercapacitor and the outputs of MPL and MSOPL. When the MPL signal is high then the SWIPT receiver controller starts the counting operation. When the voltage across the supercapacitor is increased by harvesting energy from the ambient environment, then the MSOPL signal is activated. At this point, the SWIPT controller stops the counting operation and estimates the available RF energy in the ambient environment. Similarly, the MSOPL signal is low when the SWIPT receiver controller starts the energy consumption counter. When the MPL signal is low, then it stops the counter and estimates the power consumption rate. ASK modulated signals are generated through the vector signal generator. The SSF frame modulated with 2-ASK and 4-ASK are shown in Figure 22. This consists of preamble, SYNC, data bytes and CRC. The transmitted bits are shown in hexadecimal format. The preamble is used to recover the bit sampling timing at the SWIPT receiver. SYNC is used to align the data frames and indicates the start of the data packets. The SSF frame always uses 2 bytes which contain control information for the SWIPT receiver. The 4-ASK modulated SSF is used for higher data rate transmission. The binary values of each amplitude of 4-ASK modulation are also shown. Four different amplitudes are used to encode the transmitted information. Data communication between the SWIPT transmitter and SWIPT receiver using the time switching scheme is shown in Figure 23. When the ID path is enabled, then the synchronization and data frames are exchanged between the SWIPT transmitter and the SWIPT receiver. During the energy harvesting period, the supercapacitor stores energy to perform data communication during the ID path duration. When the input RF power from the ambient environment is not sufficient, then the supercapacitor requires more energy harvesting duty ratio to have sufficient time to harvest the required energy. As we increase the duty ratio for energy harvesting, the voltage across the supercapacitor is increased. When the RF input power from the ambient environment is sufficient, then we can reduce the duty ratio for energy harvesting, which allows more time for information decoding while keeping sufficient energy stored in the supercapacitor. This condition allows us to switch to PS mode and use high-rate communication. Figure 24 shows the data communication between the SWIPT transmitter and SWIPT receiver during the TS mode, PS mode, and digital clock adjustment. This shows the effect of change in the duty ratio for energy harvesting in the TS mode on data rates. When the energy harvesting duty ratio is 35% in the TS mode, the data rate is 1 kbps with 32 kHz digital clock of the SWIPT receiver. When the duty ratio is reduced to 10%, then the data rate is increased to 2.5 kbps at a 32 kHz digital clock of the SWIPT receiver. When the RF energy is sufficient in the ambient environment for the simultaneous operation of the EH path and ID path, then the PS mode is selected. Since the EH path and ID path operate simultaneously in PS mode, this results in an increase in data rate up to 8 kbps with a digital clock of 32 kHz. After this point, if the RF energy in the ambient environment is sufficient, then the digital clock adjustment mode is selected. In this mode, the digital clock of the SWIPT receiver is adaptively increased, which results in higher data rates. Thus, data rates of 16, 32, and 256 kbps are to be achieved by increasing the digital clock by 64 kHz, 128 kHz, and 1 MHz, respectively. The proposed design of SWIPT is compared with PCB-based SWIPT implementation [36] in Table 1.

ASIC-based SWIPT design consumes very low power and area which is desirable for SWIPT operation. With the addition of ACCP, proposed SWIPT design can operate in ultra-low ambient energy while providing optimum performance. Due to similar energy harvesting and data processing structure, the comparison is done with RFID systems which are summarized in Table 2. The proposed design consumes less power as compared to other designs at 5.8 GHz. This consumes 12.3 µW which includes analog and digital blocks. The proposed design adaptively controls the clock frequency which results in adaptive data rates. The occupied area is very small as compared to other designs.

## 5. Conclusions

In this paper, an adaptive communication and control protocol is presented for ultra-low power simultaneous wireless information and power transfer (SWIPT) system operation for sensor applications. Unlike other wireless powered devices which have a dedicated source of energy, the SWIPT system depends on the available RF energy present in the ambient environment. Through the proposed protocol, the SWIPT system adapts its configuration depending on the available RF energy in the ambient environment. This adaptively improves the performance of the SWIPT system in terms of energy harvesting and data communication. The proposed protocol further allows to improve the data communication performance by adaptively increasing the digital clock frequency at the SWIPT receiver. The proposed SWIPT receiver design is implemented in 180 nm CMOS technology and consumes significantly less power (12.3 µW). This also occupies less area—0.81 mm^2^. The clock frequency can adaptively vary from 32 kHz to 2 MHz. This results in an adaptive data rate from 8 to 500 kbps.

## Figures and Tables

**Table 1:** Performance comparison with the PCB-based SWIPT.

	Sensor 2019 [36]	This Work
Technology (µm)	PCB	0.18
Architecture	SWIPT	SWIPT
Harvest source	Ambient RF	Ambient RF
Power (µW)	NA	12.3@ 2 MHz
Area (mm^2^)	NA	0.067
Clock frequency (MHz)	NA	0.032–2
Data rate (kbps)	1000–4000	8–500
RF frequency (GHz)	0.900	5.8
Protocol	NA	ACCP
Downlink modulation	ASK/BPSK/PAPR	ASK
Uplink modulation	Backscattering	Backscattering

**Table 2:** Performance comparison with RFID.

	TIE 2020 [38]	TIE 2016 [39]	JSSC 2010 [40]	This Work
Technology (µm)	0.18	0.18	0.18	0.18
Architecture	RFID	RFID	RFID	SWIPT
Harvest source	RF/VL	RF/Battery	RF	Ambient RF
Power (µW)	64	66.3	16.6	12.3@ 500 kbps
Area (mm^2^)	2.4	1.1	4.5	0.81
Clock frequency (MHz)	1.92	0.032	10	0.032–2
Data rate (kbps)	40	NA	1000	8–500
RF frequency (GHz)	0.902–0.928	0.860–0.930	5.8	5.8
Downlink modulation	ASK	ASK	ASK	ASK
Uplink modulation	Backscattering	Backscattering	Impulse OOK, BPSK	Backscattering

## Data Availability

Not applicable.
